# Ileoileal intussusception in the adult patient secondary to a fibroma: An organ-preserving approach to management

**DOI:** 10.1093/jscr/rjab253

**Published:** 2021-06-30

**Authors:** Enda Hannan, Aisling Egan, Alison Bell, Muireann Murray, Eoin Martin, Gerard Byrnes

**Affiliations:** Department of Surgery, University Hospital Limerick, Co. Limerick, Ireland; Department of Surgery, University Hospital Limerick, Co. Limerick, Ireland; Department of Surgery, University Hospital Limerick, Co. Limerick, Ireland; Department of Surgery, University Hospital Limerick, Co. Limerick, Ireland; Department of Radiology, University Hospital Limerick, Co. Limerick, Ireland; Department of Surgery, University Hospital Limerick, Co. Limerick, Ireland; School of Medicine, University of Limerick, Co. Limerick, Ireland

## Abstract

We present a rare case of adult intussusception (AI) due to a small bowel fibroma, which presented as recurrent subacute symptoms. To our knowledge, this is the first reported case managed by intraluminal excision of the causative lesion by enterotomy as opposed to bowel resection. A 34-year-old woman presented with recurrent colicky abdominal pain. Computed tomography demonstrated ileoileal intussusception, with magnetic resonance imaging revealing a 2.3-cm intraluminal lesion acting as a lead point. The patient underwent laparotomy and the intussusception was reduced. Palpation of the lesion demonstrated a pedunculated polyp without suspicious features; so the lesion was resected via enterotomy as opposed to small bowel resection. Histopathological analysis diagnosed a benign fibroma. AI is a rare but important entity, with potentially devastating consequences for delayed diagnosis. The majority of lesions causing AI are benign and may be amenable to intraluminal resection via enterotomy, thus avoiding unnecessary bowel resection.

## INTRODUCTION

Intussusception is the telescoping of one segment of intestine into an adjacent segment [1]. This can cause small bowel obstruction, perforation and mortality [1]. The condition is exceedingly rare in adults, with an incidence of less than three cases per 1000 00 population annually [1]. Most cases of paediatric intussusception (PI) are idiopathic and can be managed with reduction by contrast enema [1]. However, adult intussusception (AI) is associated with underlying pathology in 90% of cases and thus almost always requires surgical intervention [2]. The infrequency of AI means that diagnosis can be delayed, which can have serious consequences. We present a case of ileoileal AI secondary to a small bowel fibroma, an extremely rare cause of this condition with only one previously reported case in the literature [3]. This case also demonstrates an atypical clinical presentation and an organ-preserving approach to surgical management for this rare but important condition.

## CASE PRESENTATION

A 34-year-old woman presented to the emergency department with colicky abdominal pain, having presented 3 months prior with similar symptoms. At that time, she was discharged after an unremarkable abdominal ultrasound scan and gastroscopy. Her abdomen was diffusely tender throughout on this occasion, with localized guarding in the upper abdomen. Haematological and biochemical investigations revealed a raised white cell count (17 × 10^3^/μl, normal range 5–10 × 10^3^/μl) and serum lactate (2.7 mmol/l, normal range 0.5–1 mmol/l).

Abdominal and chest plain films were unremarkable. Computed tomography (CT) of the abdomen and pelvis was performed, which demonstrated an intussusception involving 12 cm of mid-ileum in the right upper quadrant. No pathological lead-point for the intussusception was identified. Magnetic resonance imaging of the small bowel was then performed, identifying a 2.3-cm intraluminal lesion within the lumen of the ileum at the lead point.

**Figure 1:**
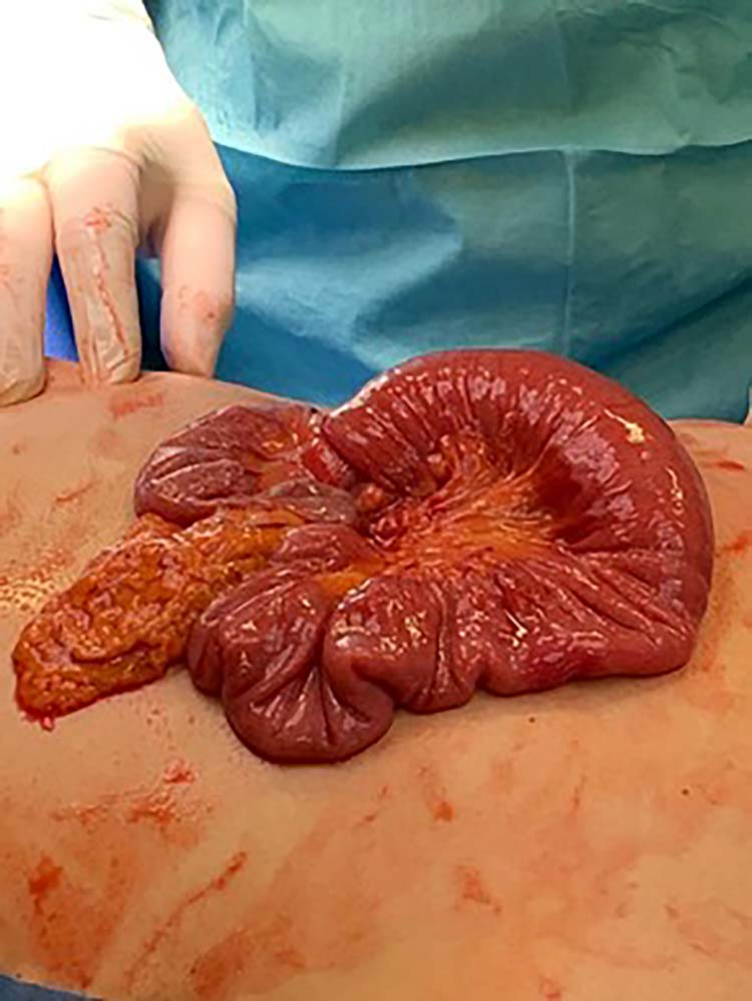
Small bowel intussusception causing small bowel obstruction.

**Figure 2:**
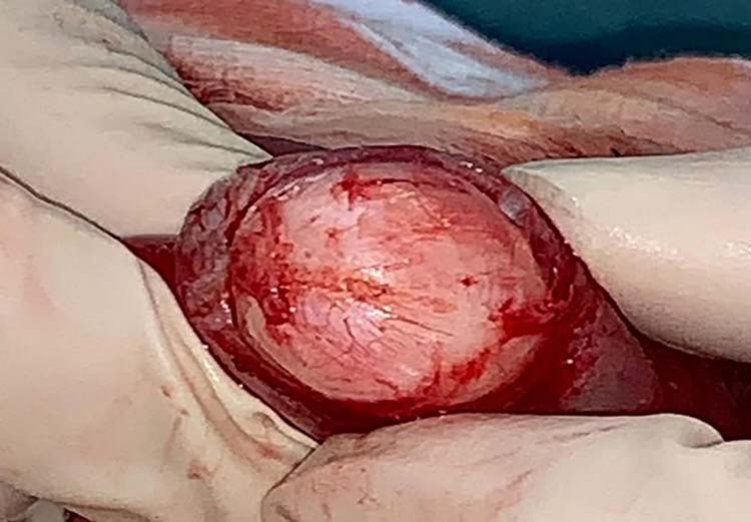
Longitudinal enterotomy with demonstration of intraluminal lesion.

**Figure 3:**
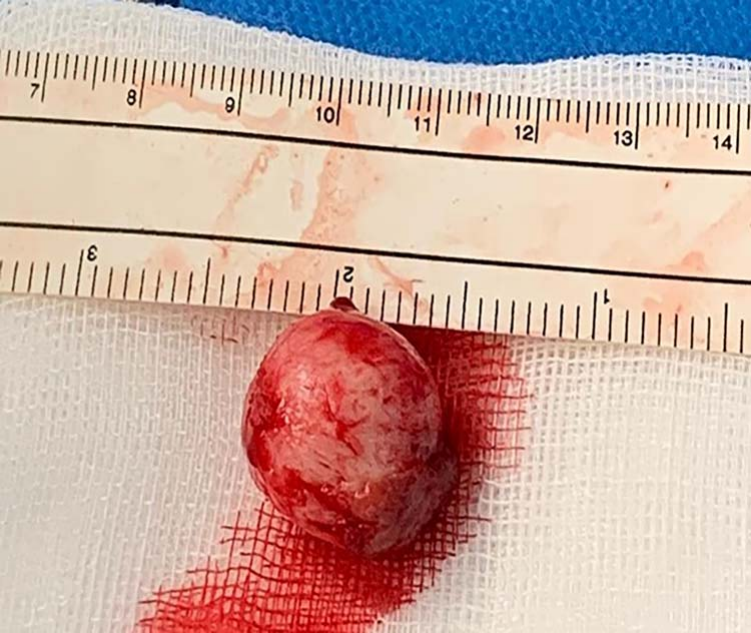
Resected intraluminal lesion.

The patient underwent an exploratory laparotomy. The intussuscepted ileum was identified and manually reduced (Fig. 1). The bowel was viable, but a small intraluminal lesion was palpable. This was smooth, rubbery, highly mobile, with a palpable stalk at its base, suggesting a pedunculated polypoidal lesion. There were no suspicious features or associated mesenteric lymphadenopathy. A longitudinal enterotomy was made over the lesion to allow further examination (Fig. 2). Given the lack of macroscopically suspicious features and its pedunculated nature, the decision was made to excise the lesion intraluminally at the base of its stalk, as opposed to performing a small bowel resection (Fig. 3). The specimen was sent for histopathological examination. The enterotomy was closed transversely using interrupted seromuscular 3.0 polydioxanone (PDS) sutures. This decision was made on the basis that, should the final histopathological report suggest suspicious or potentially malignant pathology, it would be possible to return to surgery to perform a formal small bowel resection at the marked point of enterotomy. However, it was felt intraoperatively that this was highly unlikely and that this approach would allow the patient to avoid unnecessary loss of small intestine or the risk of anastomotic dehiscence.

The patient made a prompt recovery and was discharged 7 days post-operatively. She remains well at outpatient follow-up. Histopathological examination resulted in a final diagnosis of a benign fibroma with clear margins.

## DISCUSSION

AI is a rare entity with potential for severe complications if not recognized [1]. While PI is associated with a classical triad of colicky abdominal pain, ‘currant jelly’ stool and a palpable sausage-shaped mass, AI is rarely so obvious and is often associated with multiple presentations with non-specific symptoms [1]. This may result in a potentially devastating delay in intervention, with bowel ischaemia, necrosis, perforation, peritonitis and mortality potentially resulting from a missed diagnosis of intussusception [1]. This case serves to exemplify the subacute clinical presentation often seen in AI. The presentation 3 months prior likely represented incomplete small bowel obstruction with subsequent spontaneous resolution. Intermittent crampy abdominal pain is the most common symptom in AI, with <20% initially presenting with an acute abdomen [4]. The presence of a causative underlying lesion in most adult patients means that recurrence is highly likely, and thus, surgery will be necessary [3]. Given the rarity of AI and the absence of obvious clinical features, the diagnosis is often made radiologically [5]. Our case serves to highlight the importance of thoroughly investigating patients with recurrent gastrointestinal symptoms by cross-sectional imaging when their symptoms have not been satisfactorily explained by initial investigations, even in young patients where malignant pathology is felt to be unlikely. Recurrent presentations with crampy abdominal pain in adults unexplained by endoscopy or ultrasonography should raise suspicion of small bowel pathology, such as intussusception, which can be diagnosed on CT with a sensitivity of up to 87.5% and specificity of 100% [5].

The vast majority of reported cases of AI advocate for surgical management [1–5]. This is due to the high likelihood of an underlying lesion acting as a lead point, which necessitates removal to prevent recurrence of intussusception [1, 2]. However, in almost all cases, a small bowel resection was performed, even in the context of viable bowel, despite most yielding benign pathology [1–5]. Malignant small bowel tumors are rare, with small bowel AI most commonly being a result of benign lesions, such as lipomas, Peutz–Jeghers polyps and adenomatous polyps [5]. To our knowledge, our case is the first in the literature where an organ-preserving approach to lesion removal was used in AI, whereby an intraluminal lesion without suspicious features was removed via enterotomy. This allowed the patient to have the lesion excised, thus preventing further intussusception, while also avoiding an unnecessary small bowel resection for likely benign disease. Such an approach may be employed on a case-by-case basis for AI and in the context of a lesion without suspicious features identified radiologically or intraoperatively, allow the patient to avoid the risks associated with small bowel resection and anastomosis, such as anastomotic dehiscence, internal herniation and post-operative small bowel obstruction. Factors in this case that advocated for such an approach were the pedunculated nature of lesion, which allowed for intraluminal excision without significant defect or stenosis and an intraoperative examination that suggested benign pathology. Small bowel fibroma is an extremely rare cause of intussusception, with only one previously reported case in the literature [3].

## CONFLICTS OF INTEREST STATEMENT

None declared.

## FUNDING

None.

## CONSENT

Obtained from the patient and documented in the medical notes.
